# Editorial: New Models of Care for Patients with Severe Mental Illness—Bridging In- and Outpatients

**DOI:** 10.3389/fpsyt.2018.00003

**Published:** 2018-01-26

**Authors:** Alexandre Wullschleger, Yasser Khazaal, Martin Heinze

**Affiliations:** ^1^Charité Universitätsmedizin Berlin, Berlin, Germany; ^2^Geneva University Hospitals (HUG), Genève, Switzerland; ^3^Immanuel Klinik Rüdersdorf, Rüdersdorf, Germany; ^4^Brandenburg Medical School Theodor Fontane, Neuruppin, Germany

**Keywords:** models of care, case management, severe mental illness, outpatient treatment, integrated care

**Editorial on the Research Topic**

**New Models of Care for Patients with Severe Mental Illness—Bridging in- and Outpatients**

In accordance with the concept of recovery (1, 2) and since the UN convention on the rights of persons with disabilities (3), psychiatric services are compelled to provide comprehensive and flexible care to persons suffering from severe and chronic psychiatric disorders, in order to help them overcome the barriers they meet, and support their full and effective participation and inclusion in society. This requires a high level of cooperation between different sectors and care providers working in this field, most notably between the in- and outpatient sectors. Unfortunately, most countries and institutions are characterized by a pervasive fragmentation of services and a lack of integration of different treatment and support options. Because of these insufficiencies, many severely ill patients cannot benefit from the comprehensive care they need. These patients often show high rates of treatment drop-outs and comorbid disorders. They are, notably, more frequently unemployed and often receive long-term disability. Although these unfavorable and complicated courses of disease have been mostly accepted as normal in the past, there nowadays is no doubt that persons suffering from severe mental illnesses (SMIs) can also fully recover and that the quality and organization of the mental health services plays a central role in this process (4).

Psychiatric services must, therefore, ensure that the most severely ill patients have access to high-quality care. As a response, many new models aiming at bridging existing services and offering need-adapted, flexible psychiatric care have been developed over the last years across Europe. On the institutional level, they encompass strategies to evaluate the needs of service users, transitional models to provide intensive support after hospital stays as well as outreach and assertive outpatient models. All these models need to adapt to the local particularities of the care system and tailor their interventions to reach all patients including those suffering from severe conditions. As for the non-institutional level, they aim at developing new practices that encourage and support individual recovery through all sectors of care, and that prevent to a maximum the use of interventions such as informal coercion hindering patients in their self-determination.

Hence, the aim of this Research Topic is to reflect on new models of care and compare how the institutionally difficult question of combing in- and outpatient-service is solved in the psychiatric care systems in different European countries. All the contributions to this topic underline different aspects of this problem and should stimulate further research and debate.

In their systematic review, Morin et al. show that many rehabilitative interventions such as cognitive remediation, psycho-education, or social skills training have a positive effect on promoting recovery for people suffering from schizophrenia and thus argue for a greater integration of these interventions in all sectors of psychiatric services to reach all concerned patients. In a similar way, Hotzy et al., in their systematic review, advocate about informal coercion for a stronger cooperation between services, to ensure that more attention is warranted for this subject and to allow clinical and ethical guidelines to be used when applying informal coercion in all sectors of care. Only in this way can the assumed negative effects of informal coercion such as increased stigma, impairment of the therapeutic relationship, and avoidance of mental health care be prevented.

The assessment of patients’ needs is also a central issue in the development and implementation of new models of care. In their review article, Zaninotto et al. underline the need to thoroughly assess psychiatric conditions in patients who suffered from traumatic brain injury in order to provide adapted treatment contributing to their full recovery and coordinate rehabilitative interventions. In a contribution dedicated to the evaluation of integrative models of care in Germany, Ignatyev et al. present a new scale aiming at including the views and opinions of patients in the evaluation of new models of integrative care. This evaluation should contribute to taking this essential perspective into account while implementing and developing such projects.

Case management also receives particular attention in this topic as a way of bridging in- and outpatient care. Penzenstadler et al. in a systematic review report on the possible effects of case management for people suffering from substance use disorders. They show that case management can positively influence global functioning and adherence to treatment, thus underlining the importance of such models linking different care sectors. Beside this review, two original research articles studied the effects of transitional case management (TCM) after inpatient stay. Bonsack et al., in their contribution, describe a positive effect of TCM on short-term engagement in outpatient care, but no effect of such an intervention in readmission rates. Similarly, Hengartner et al. showed, in their study, that a short-term TCM led to no effect on readmission rates, psychopathology, and quality of life. Both articles argue that TCM is probably most effective for people suffering from SMIs and advocate for specific interventions designed to reach this particular patients group.

Finally, the issue of integrative care models as implemented in Germany over the last years is addressed in two articles. Wullschleger et al. report on the effects of the implementation of such a model linking in- and outpatient sectors. Although the model reached the most severely ill patients, it failed to lead to the expected decrease in the average length of hospital stays, thus underlining the difficulties inherent to the implementation of new models of care. As for the second article, Mayer-Amberg et al. show that a new model of care, the Integrative Care Initiative Schizophrenia, led to a significant reduction of the duration of hospital stays and to high patient satisfaction, thus proving that such models can be successfully implemented.

All these contributions show how vast and complex the issue of enhancing the cooperation between different sectors of care is, in order to provide comprehensive help to the most severely ill patients. They call for further research and discussion about the best ways of overcoming the obstacles and barriers that unfortunately too often characterize psychiatric services.

## Author Contributions

AW wrote the main part of the editorial. YK and MH reviewed the manuscript and added significant modifications.

## Conflict of Interest Statement

The authors declare that the research was conducted in the absence of any commercial or financial relationships that could be construed as a potential conflict of interest.
